# Facts in counterfactuals-cognitive representations of Chinese counterfactuals

**DOI:** 10.1038/s41598-023-49775-x

**Published:** 2023-12-27

**Authors:** Yufei Ren, Gang Cui, Stella Christie

**Affiliations:** 1 Department of Foreign Languages and Literatures, Tsinghua University, Beijing, China; 2 Department of Psychology, Tsinghua University, Beijing, China; 3 Tsinghua Laboratory of Brain and Intelligence, Tsinghua University, Beijing, China

**Keywords:** Psychology, Human behaviour

## Abstract

How do people represent counterfactuals? As languages differ in expressibility of counterfactuals—some languages employ explicit grammatical marking for counterfactuals while others do not—are some speakers’ representations of counterfactuals less explicit? Prior studies examining this question with Chinese speakers—a language devoid of explicit counterfactual markings—found mixed results. Here we re-examined the issue by using a more sensitive test: people’s sensitivity to detect anomalies in sentences. We asked Chinese speakers to rate the acceptability of sentences employing *“ruguo (if)…jiu (then)”* configuration—the typical but non-unique, non-explicit marking of counterfactuals. Critically, we varied the semantic adherence to real-world facts [factuality], with some sentences containing made-up conditions [-fact as in *“If fish had legs, then…”*] versus real facts [+ fact: *“If dogs had legs, then…”*]. If speakers represent counterfactuals clearly, they should give higher acceptability ratings to [− facts] than to [+ facts] sentences, because the ostensible point of counterfactuals is to express non-factual situations. That is, expressing a true fact under a syntactic counterfactual construction makes the sentence anomalous. Instead, we found that Chinese speakers gave the opposite ratings: factual “*if…then*” sentences were rated as more acceptable than non-factual ones. This suggests that Chinese speakers find the processing of counterfactuals to be more challenging than processing facts, and that their representation of counterfactuals may be less explicit. Overall, this research contributes to our understanding of the link between linguistic markings and cognitive representations.

## Introduction

Counterfactual thinking, a cognitive process marked by reflections on “what might have been”, is pervasively used in everyday reasoning^1,2^. People engage in counterfactual thinking in order to imagine alternative realities or outcomes^3–5^, a fundamental reasoning for planning and prediction, as well as for expressing emotions like regret and relief^6,7^. Even young children use counterfactuals, for example when doing pretend play^8^. There is evidence that counterfactual reasoning is instrumental in fostering an understanding of causal relationships—what causes a particular event to happen^9,10^ —as well as to aid acquisitions of theory of mind^11–13^, a critical skill that enables individuals to attribute mental states to others.

Interestingly, despite the pervasiveness of counterfactual reasoning, not all languages have explicit syntactic markings for counterfactuals. Some languages do mark counterfactuals using distinct morphological markers, such as subjunctive moods, past tenses, imperfective aspects, perfective aspects, or an integrated blend of tense and aspect^14–16^. For example, in English, the sentence ‘Had today was Sunday, I would have been at home’ explicitly marked a contrary-to-fact situation that today is not Sunday.

But some languages like Chinese do not have explicit counterfactual markings. Unlike the explicit morphological markers in Indo-European languages, the conveyance of counterfactual meanings in Chinese relies on a combination of contextual and lexical cues. These cues include elements such as temporal indicators, negation, the intonation marker ‘*le*’, and a diverse array of hypothetical conjunctions^17–19^. But because the counterfactual cues are neither explicit nor unique—the same cues can be used to convey other things, such as conditionals—there is an ongoing debate of whether and how Chinese speakers process and represent counterfactuals. For example, Bloom (1981)^20^ posited that the absence of counterfactual linguistic markers in Mandarin leads to a deficit in counterfactual thinking among its speakers. However, subsequent research on Chinese counterfactuals^21,22^ counters this view, suggesting that Mandarin speakers are, in fact, adept at comprehending counterfactuals. Similarly, Li and Thompson^23^, Eifring^24^, and Wu^25^ argued that while Chinese may lack inflectional means to denote counterfactuality, lexical and contextual tools facilitate Chinese speakers’ understanding of counterfactuals. But yet another study by Yeh and Gentner^26^ showed that while Chinese speakers can judge a counterfactual nature using world knowledge, such as “*If antibiotics were never invented…,*” they had difficulties to judge counterfactual assertions based on contextual knowledge.

Here we want to revisit the issue by investigating the relationship between linguistic marking and cognitive representations that it affords. Different from prior research focusing on Chinese speakers’ accuracy in comprehending counterfactual stories or statements, we instead ask whether linguistic expressions denoting counterfactuals are deemed acceptable by speakers. Using acceptability judgment, as opposed to comprehension accuracy, gives a more complete picture of speakers’ mental representation as a whole—what they consistently and habitually represent, as opposed to task-taking ability^27–30^. Indeed, people give unambiguous and consistent acceptability ratings for a vast majority of linguistic stimuli^31^, making acceptability judgment a valid tool to understand humans’ repertoire of linguistic and cognitive representations. Studies using acceptability judgment also reveal certain constructions, such as center embeddings, are often judged unacceptable albeit being grammatically sound^32^, while other instances of grammaticality illusions are deemed moderately acceptable notwithstanding their ungrammaticality and uninterpretability^33^. In sum, people’s judgment of acceptability can reflect their true mental representations.

To probe people’s mental representation of counterfactual sentences, we are particularly interested in seeing how people accept (or not)—abnormalities in sentence constructions—whether syntactic or semantic^34–37^. Syntactic anomalies usually stem from the absence of grammatical markers or the violation of syntactic rules like word order, while semantic anomalies often appear as contradictions or conflicts with accepted reality. For instance, the sentence, “*A widow is talking to her husband*” exhibits semantic anomaly given societal understanding of a “*widow”* precludes the presence of a husband.

But in the context of counterfactual sentences, conflicts with accepted reality ought *not* be judged as semantic anomalies. This is because counterfactuals precisely express alternative realities—conflicts with reality should in fact be expressed in counterfactuals. The opposite—using counterfactuals to express real facts—would be odd; imagine saying to your own child “*If only you were my child, I would have named you to be my heir*”. Indeed, studies have found that while violation of facts are deemed semantically anomalous, within counterfactual settings, they are deemed acceptable^38,39^. For example, Nieuwland and Van Berkum^38^ discovered that the N400 effect dissipated when an ostensibly impossible event (e.g., the peanut fell in love) was contextualized within a counterfactual setting. Our question is whether Chinese speakers share this intuition.

To create semantic anomalies in counterfactual sentences, we need to use the typical linguistic markings for counterfactuals in Chinese. While Mandarin lacks tense markers similar to the subjunctive mood for explicitly delineating counterfactuals, it often employs specific connectives, notably the* “ruguo(if)…jiu(then)”* structure^23,40,41^. These connectives adhere to a relatively fixed syntactic pairing, a concept introduced early in educational settings. The rationale is that if this typical *“ruguo(if)…jiu(then)”* syntactic framework easily evoke counterfactual representations, then we expect speakers to rate *non-factual* antecedent, such as the sentence “*If fish had legs, then…*” to be acceptable. Likewise, speakers should rate factual antecedent sentences, such as “*If dog had legs, then…*” to be less acceptable (anomalous). On the other hand, if the construction* “ruguo(if)…jiu(then)”* does not evoke explicit counterfactual processing, then speakers will not differentiate between these two types of sentences, or they may just default to facts in judging acceptability—true facts are judged acceptable, while non-true facts are not.

## Methods

### Design

Counterfactuals broadly bifurcate into two primary categories based on the nature of their falsified subjects. The first category concerns falsified past events and is frequently used to convey sentiments of regret, rumination, or disguise. For instance, in the sentence, “*If I had studied harder, I would have passed the exam”,* conveys a sense of regret over not having studied adequately. The second category pertains to falsifications of the physical world, where the premise contradicts established physical laws. An example sentence, “*If a lion could speak”*^39^, contravenes the known fact that lions do not have the capacity for speech. In this study, we focused on the second type of counterfactuals to create semantic anomalies, as they can afford clearer violations of facts.

To compare speakers’ acceptability to anomalous vs. non anomalous sentences in counterfactual vs. non-counterfactual setting, we created 4 types of sentences using two parameters: syntactic pairing [+ /− syntax] and factuality [+ /− facts]. For the canonical syntactic pairing [+ syntax], we used the *“ruguo (if)…jiu (then)”* construction. This pairing is deeply entrenched in the Chinese language, forming an integral part of linguistic instruction from primary education onwards. The *“jiu (then)”* particle, when paired with *“ruguo (if)”* serves to emphasize the consequential nature of the hypothetical scenario introduced by *“ruguo (if)*”—in other words, it is a construction that ought to elicit counterfactual reasoning. For the illicit syntactic pairing [− syntax] we used the *“ruguo (if)…suoyi (so),”* a configuration that diverges from conventional Chinese syntax. There is logical incongruity between *“ruguo (if)”* and *“suoyi (so)”*: while* “ruguo (if)”* lays the foundation for a hypothesis, be it conditional or counterfactual, the particle “*suoyi (so)”* serves as a marker of causal reasoning which requires a factual premise rather than a hypothetical one^23^. True fact [+ facts] sentences contain true assertions about animals’ attributes [e.g., dogs have legs], while [− facts] sentences contain non-true attributes of animals (e.g., fish have legs). We chose animals’ attributes so there are no ambiguities of what constitute as true vs. non-true facts.

These two parameters created 4 types of test sentences (a 2 × 2 design): [+ syntax − facts]; [+ syntax + facts]; [− syntax + facts]; [− syntax + facts]. The most critical comparison is between [+ syntax − facts] vs. [+ syntax + facts] sentences. Type 1 sentences [+ syntax − facts] denote non-anomalous counterfactuals because they express non-true facts under a syntactic framework of hypotheticals. On the other hand, Type 2 [+ syntax + facts] sentences are semantically anomalous, as they expressed true facts using a syntactic pairing for hypotheticals. If *“ruguo (if)…jiu (then)”* construction evokes counterfactual reasoning, speakers should give higher acceptability ratings to [+ syntax − facts] sentences than to [+ syntax + facts] ones. On the other hand, if speakers mostly care about factuality (rather than to counterfactuality), they might give higher ratings to all true-fact sentences ([+ syntax + facts] and [− syntax + facts]) compared to the [-fact] sentences. As a secondary interest, we also wanted to investigate Chinese speakers’ grammatical representations, comparing the canonical syntactic pairing [+ syntax + /− facts] sentences with the illicit syntactic pairing sentences [− syntax + /− facts]. In total, there were 24 unique test sentences, 6 sentences per type (see Table 1 for the design and example sentences).

**Table 1:** 2 × 2 design of test sentences and examples.

Parameter	[+ factuality]	[− factuality]
[+ Syntactic pairing]	[+ syntax + facts] ① *ruguo gou you tui jiu keyi pao* if dogs have legs then can run If dogs have legs, then they can run	[+ syntax − facts] ② *ruguo yu you tui jiu keyi pao* if fish have legs then can run If fish had legs, then they could run
[- Syntactic pairing]	[− syntax + facts] ③ *ruguo gou you tui suoyi keyi pao* If dogs have legs so can run If dogs have legs, so they can run	[− syntax − facts] ④ *ruguo yu you tui suoyi keyi pao* If fish have legs so can run If fish had legs, so they could run

In addition to test sentences, we created filler sentences containing the same typical and illicit syntactic pairing used in test sentences (*“ruguo (if)…jiu (then)”* and *“ruguo (if)…suoyi (so)”*), but now with conditional statements. Examples of filler statements are “*Ruguo tianqi hao jiu keyi pa shan” (If the weather is good, then (we) can climb a mountain*.); or “*Ruguo chengji hao suoyi neng shou biaoyang” (If grade is good, so can get a praise)*. The filler sentences serve several purposes: (i) to create variability so participants are not forming any particular expectations about the nature of the experiment, (ii) to maintain attention and engagement during the study—the filler sentences use interesting, varied everyday situations (compared to the test sentences that contain animal attributes only), and (iii) to serve as comparison baseline to the test sentences. We expected participants to find the filler sentences acceptable, as they are conditional statements without violation of facts. This rating serves as a baseline of what constitutes as high acceptability ratings.

### Participants

300 native speakers of Mandarin Chinese participated in this experiment. Participants were middle school educators from the Xinjiang province of China and they represented a broad spectrum of age demographics and academic training background. Teachers’ specialization includes Chinese, English, Mathematics, and an assortment of Sciences. We ran the study with middle school teachers as they were a convenient sample at the time of the study, but also because we expected that teachers—more than ordinary individuals—might have a more explicit command of Chinese grammars and hence, provides a more stringent test to our question.

The final data set of the study includes responses from 262 individuals. This sample size was determined after excluding participants who did not complete the task.

### Procedure

Participants were asked to rate sentences’ acceptability using a Likert scale ranging from 1 (not acceptable at all) to 7 (very acceptable). The instruction for the rating task emphasized that participants should give judgements based on their own understanding; there is no right or wrong answer.

Sentences were presented one-by-one in a counterbalanced sequence, ensuring that no two consecutive sentences belonged to the same category. Participants judged a total of 36 sentences (24 test sentences and 12 filler sentences). All participants did the task on their mobile phones within a designated time frame during an official forum convened by the Education Authority of Heshuo County, Xinjiang Province, China. The formal setting of the forum may have exerted an influence on the participants’ diligent engagement with the assigned task.

This study was conducted in strict adherence to ethical standards, with approval from the Education Authority of Heshuo County’s Institutional Review Board. Informed consent was obtained from all participants, ensuring their participation was voluntary and their data confidential. Participant information was anonymized to maintain privacy. The methods used were in compliance with the guidelines and regulations of the conference, and the experimental protocols received approval from Tsinghua University.

### Analysis

Utilizing G*Power 3.1, we calculated an a priori sample size of 36 for repeated measures within factors to achieve 0.95 statistical power. Consequently, our actual sample size of 262 participants is more than sufficient and statistically robust. To compare filler and experimental items initially, we employed a *t*-test to determine the baseline acceptability of conditional sentences. Following this, we performed a factorial analysis of variance (ANOVA) on z-scores from participants’ acceptability ratings to evaluate the effects of [Factuality] and [Syntactic Pairing] on sentence acceptability. The analysis, along with the generation of corresponding data and graphical representations, was performed using Python v2023.20.0, specifically employing the matplotlib and statsmodels packages.

## Results

As the study encompassed four distinct sentence types: [+ syntax + facts], [+ syntax − facts], [− syntax + facts], and [− syntax − facts], we first checked whether these 4 types indeed form consistent categories. A reliability analysis yielded high Cronbach’s alpha values for all 4 types: 0.8514 ([+ syntax + facts]), 0.8469 ([+ syntax − facts]), 0.8346 ([− syntax + facts]), 0.8471 ([− syntax − facts]). Analysis of the two types of filler sentences also yielded high Crobach’s alpha: 0.8757 (conditional [+ syntax]) and 0.7457 (conditional [− syntax]). Overall, the results suggest that there is good to excellent internal consistency across all categories of sentences.

**Figure 1:**
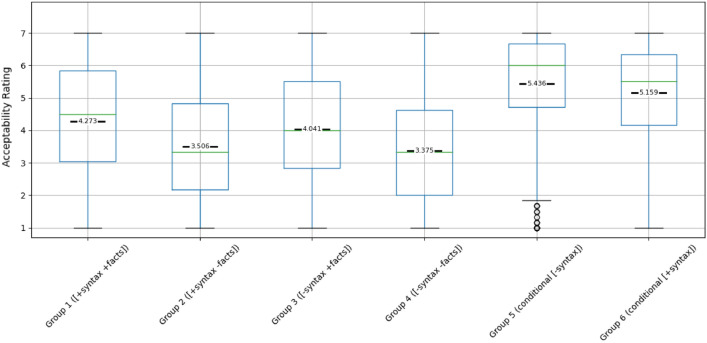
Box plot of acceptability ratings for 6 Groups (mean marked in black).

Boxplots showing means of acceptability ratings for 4 types of test sentences and 2 types of filler sentences are shown on Fig. 1. For the comparison of filler and experimental items, we first compared our critical test sentence [+ syntax − facts] with the filler conditional [+ syntax]. A *t*-test revealed that speakers found counterfactual sentences [+ syntax − facts] (Mean = 3.506) to be less acceptable than conditional [+ syntax] sentences (mean = 5.159), with *t*(261) =  − 15.10, *p* < 0.001). This finding indicates that while conditional sentences, serving as a baseline, are generally viewed as more acceptable, the experimental counterfactual sentences receive lower ratings. The large effect size, Cohen’s *d* = − 0.9326, reinforces this conclusion. These results suggest that despite the theoretical flexibility of the *“ruguo(if)…jiu(then)”* construction to express both counterfactuals and conditionals, in practice, it is predominantly used and perceived as more suitable for conditional statements.

To examine the overall impacts of [Factuality] and [Syntactic Pairing] on the acceptability of sentences, we employed a factorial analysis of variance (ANOVA) on the z-scores of participants’ acceptability ratings. Levene’s test verified homogeneity of variance across conditions (*p*-value = 0.8301, > 0.05), satisfying the prerequisite for conducting ANOVA (see Table 2).

**Table 2:** Two-way ANOVA results on sentence acceptability ratings.

	Sum_sq	df	F	PR(> F)
Factuality	70.12762	1	142.1289	8.77E−31
Syntactic pairing	4.438328	1	8.995236	0.002772
Factuality: syntactic pairing	0.068694	1	0.139223	0.709132
Residual	507.2242	1028		

Factorial ANOVA revealed a significant main effect of [Factuality] on sentence acceptability, *F*(1,1028) = 142.13, *p* < 0.0001, signifying that factual content was rated distinctly from non-factual content. The [Factuality] effect is seen through two comparison groups: [+ syntax − fact] vs. [+ syntax + fact], and [− syntax + facts] vs. [− syntax − facts]. The comparison between [+ syntax − fact] and [+ syntax + fact] is our critical comparison. Recall that our hypothesis is that speakers should give higher ratings to [+ syntax − fact] because the combination of non-true facts and *“ruguo(if)…jiu(then)”* construction satisfies all requirements for counterfactual statements. But instead, we found the opposite effect: Chinese speakers gave higher acceptability ratings to [+ syntax + facts] sentences (mean = 4.273) as opposed to [+ syntax − fact] sentences (mean = 3.506). The comparison between factual [− syntax + facts] (mean = 4.041) with the non-factual [− syntax − facts] (mean = 3.375) shows factuality matters even under illicit syntactic pairing. This result suggests that the *“ruguo(if)…jiu(then)”* construction, while typically thought to be the most common construction for expressing counterfactual thoughts, does not in fact easily evoke counterfactual processing. Instead, it seems that the most salient thing for speakers’ is truthiness of facts.

Our secondary interest is to probe speakers’ internal representations of grammar: how much of a violation is it to read new syntactic pairings, as in the *“ruguo(if)…suoyi(so)”* construction. We approached this by comparing two distinct sets of syntactic pairings: [+ syntax − facts] (mean = 3.506) versus [− syntax − facts] (mean = 3.375), and [+ syntax + facts] (mean = 4.273) versus [− syntax + facts] (mean = 4.041). Although the effect observed in these comparisons was less pronounced, it was statistically significant, as evidenced *F*(1,1028) = 8.99, *p* = 0.0028. This result indicates that the syntactic arrangement within a sentence significantly impacts how participants perceive its acceptability. This finding underscores the importance of syntactic structure in the cognitive processing of language, suggesting that even subtle changes in syntactic pairing can influence the perceived grammaticality of a sentence.

However, contrary to our hypothesis, there is no significant interaction between [Factuality] and [Syntactic Pairing], *F*(1,1028) = 0.14, *p* = 0.7091. That is, the influence of [Factuality] on acceptability ratings is not contingent upon the syntactic structure. These two variables seem to operate independently in shaping sentence acceptability, with [Factuality] making a more substantial contribution than [Syntactic Pairing]. Particularly pertinent to our question about representation of counterfactuals among Chinese speakers, the results suggest that the typical counterfactual syntactic construction does not, in fact, easily evoke counterfactual representations.

**Figure 2:**
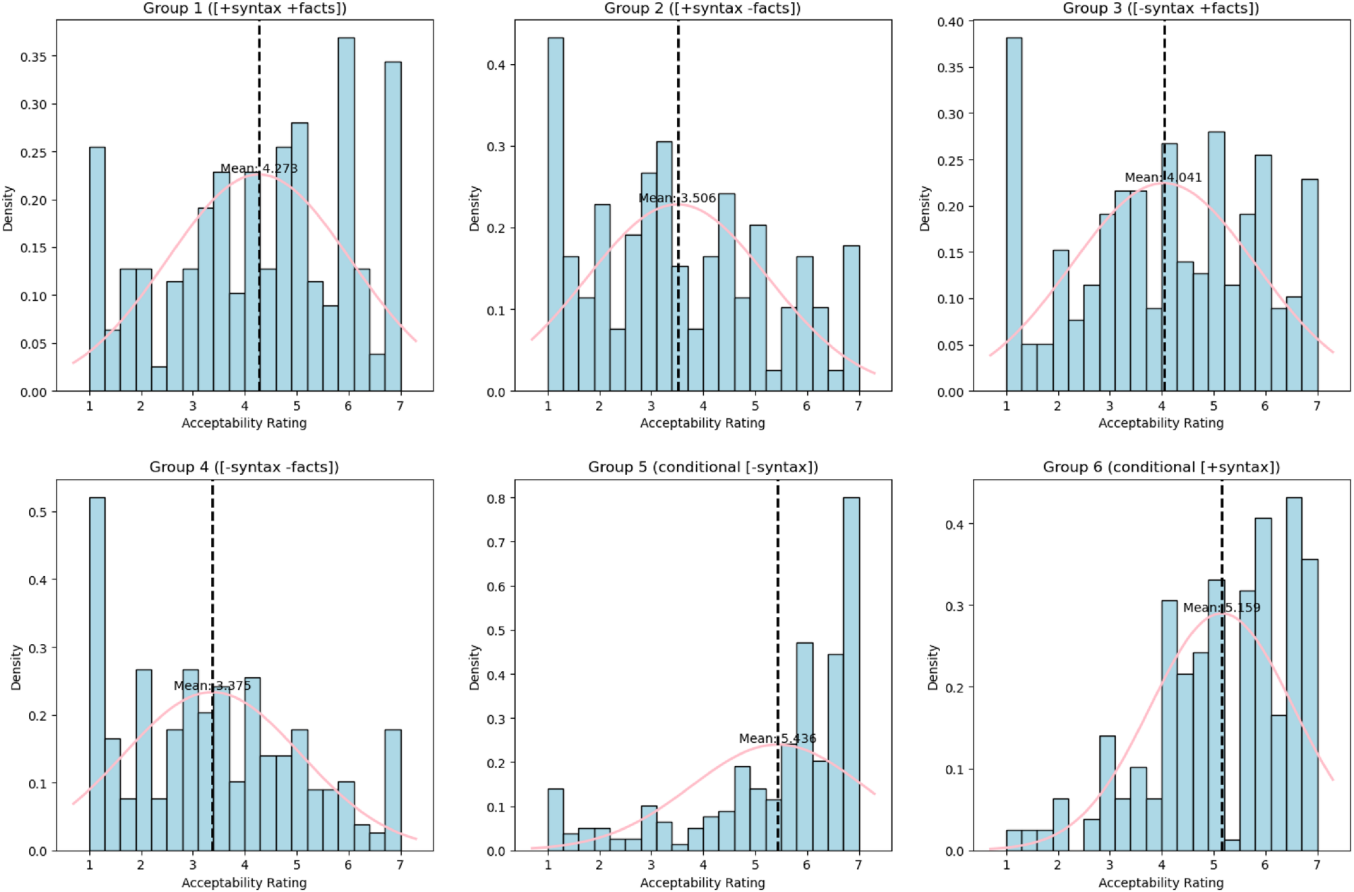
Histograms illustrating the frequency distribution of acceptability ratings across the six types of sentences.

Last but not least, to give a full picture of the distribution of participants’ ratings for each type of sentences, we included Fig. 2.

## Discussion

This study probes the processing of counterfactual constructions in Mandarin Chinese, focusing on the prevalent though non-exclusive *“ruguo(if)…jiu(then)”* syntactic pattern. Different from prior studies that measure Chinese speakers’ accuracies in comprehending counterfactual stories or statements, we instead reverse the investigative angle, asking whether a typical Chinese counterfactual syntactic construction indeed evokes counterfactual processing. Traditionally, counterfactuals are expected to score higher in acceptability when they express conditions contrary to fact, given that the purpose of counterfactuals are precisely to speculate beyond the scope of reality. Contrary to this expectation, our findings reveal that Chinese speakers rate sentences consistent with [+ facts] as more acceptable than those positing fictitious scenarios, underscoring a tendency to favor fact even within a hypothetical context. Our investigation suggest that Chinese speakers navigate sentences offering alternative realities with discernible difficulties. While engaging with counterfactuals, Chinese speakers might rely heavily on real-world factual knowledge, even in the face of linguistically presented hypothetical information.

This inclination implies that for Chinese speakers, representing counterfactuals may involve less explicit cognitive constructs, possibly because Mandarin lacks specific grammatical devices for clearly demarcating counterfactual statements. This could render the cognitive processing of such sentences more onerous, aligning with previous studies^42–44^ that found counterfactual plausibility to be enhanced by closeness to reality.

Does this mean that Chinese speakers are unable or less likely to think counterfactually? Most likely not, or at least this is not what the study design and results point to. For one, preference for true facts over non-facts does not necessarily imply that one cannot represent counterfactuals. Clearly, Chinese speakers think about alternative realities, whether in planning, expressing regrets, or determining causality of events. Rather, we think these results suggest that unlike for speakers of languages with explicit counterfactual markings, speakers of languages like Chinese do not have a default linguistic mean for expressing counterfactuals—several constructions can be used to denote counterfactuals. While linguistic analysis suggests that “*ruguo(if)…jiu(then)”* is the typical construction for counterfactuals, our results suggest that the link between this grammatical construction and counterfactual representation in the mind is less direct. Such mapping between syntactic construction and semantic representation—from direct to less direct—may be one useful way of classifying counterfactuality in language and mind. For example, English speakers most likely directly maps the past perfective syntax (*“Had I started earlier I would have finished on time”*) to a contrary-to-facts semantic representation—that the sentence’s author did in fact start late, rather than early. This direct mapping can be intuitively checked using the test we devised here: it would be odd to express true fact using counterfactual syntax, as in saying to your own (true) child “*If only you were my child, I would have made you my heir.*” But for Chinese speakers, the mapping between syntactic construction and semantic representation is less direct. Our results show that speakers do not directly map “*ruguo(if)… jiu(then)”* to semantic representation of counterfactuality.

Does direct vs. non-direct mapping between syntax and internal semantic representation impact everyday communication? One possibility is that regardless of direct or non-direct mapping, speakers of all languages are equally good in expressing counterfactuality. As reviewed before, while Chinese lack inflectional means to denote counterfactuals, there exist other lexical and contextual tools for doing so^23–25^. That is, Chinese speakers have options for how they can express counterfactuals.

The present study, while offering valuable insights, is not without limitations. Firstly, the scope of the study was confined to a single grammatical construction—the commonly used *“ruguo…jiu”* structure in Mandarin Chinese. This construction is adept at eliciting both counterfactual and conditional responses and is a prevalent feature in Chinese linguistic education from primary school onwards. However, the applicability of our findings to other counterfactual constructions remains unexplored. Future research could extend this work by investigating other counterfactual constructions, such as those employing the *“jiaru”* marker, which signifies a hypothetical premise. Such research could provide a more comprehensive understanding of the representation of counterfactuals in Mandarin.

Another notable limitation is the absence of a cross-cultural comparison employing an identical experimental paradigm. Prior studies in English, a language that explicitly marks counterfactuals, suggest that implausible scenarios within counterfactual settings are processed as acceptable during online comprehension^39^. Conducting a parallel judgment study in English or another language with explicit counterfactual marking could potentially reinforce and extend the findings of the current study. However, it is important to note that languages with explicit counterfactual marking typically do not facilitate factual antecedents in counterfactual constructions. This discrepancy prompted our focus on the Chinese language, which offers greater flexibility in this respect. The underlying cognitive and neurological mechanisms driving the observed preference for factual scenarios in Mandarin speakers, and their rejection of counter-to-fact ‘counterfactuals’, remain an open avenue for further research. Future studies employing methodologies such as eye-tracking or electroencephalography (EEG) could illuminate the intricate processes underpinning counterfactual reasoning and its neural correlates.

But returning to the potential impact of direct vs. non-direct mapping on everyday communications, while ease of expression of counterfactuals may not differ, speakers of languages with less-direct mapping may have less fluent understanding of others’ counterfactual expressions and/or intentions. This is because when speaker A chooses to use construction 1 to express counterfactuals, listener B may not map construction 1 directly to counterfactual representation, but instead to other semantic meaning, for example, conditionals. Testing this hypothesis is beyond the scope of the current study, but it would very interesting for future studies to investigate group, rather than individual sentence processing about counterfactuals. Since language is primarily a tool of social communication^45,46^, it makes sense to ask whether languages’ diverse ways of marking counterfactuals—some with direct mapping and some less direct—impact social-group communications involving counterfactuals.

## Conclusion

This research delves into Chinese counterfactuals, specifically focusing on the “*ruguo…jiu”* structure, offering a significant contribution to typological language studies and the broader domain of language processing. By analyzing the *“ruguo…jiu”* configuration within the realm of manipulated syntactic and semantic anomalies, it becomes evident that [+ facts] scenarios are preferred in terms of acceptability, irrespective of their syntactic alignment. This suggests speakers’ inherent inclination towards factual constructs, even within counterfactual contexts. Chinese speakers seem to have a cognitive bias toward factual veracity, suggesting that factual information is processed more seamlessly than counterfactual suppositions in Chinese sentence comprehension.

This orientation toward fact over hypothetical construction suggests that the mental parsing of counterfactuals in Chinese may necessitate a deliberate, more cognitively taxing process. But as discussed above, this extra effort may only apply in the case of understanding others’ counterfactuals assertions, not in individuals expressing counterfactuals. Overall this study contributes to the wider conversation in linguistic and psychological research by delineating how different facets of language—semantics and syntax—converge to shape sentence interpretation.

## Data Availability

The datasets used and/or analyzed during the current study available from the corresponding author on reasonable request.
